# Endobronchial metastasis secondary to occulting renal cell carcinoma: literature review and a rare case report

**DOI:** 10.1186/s12890-023-02320-y

**Published:** 2023-01-19

**Authors:** Seif-Aldin Abdul Rahman, Ali Abdul Rahman, Samer Rajab, Somar Mansour, Marah Mansour, Elias Salloum, Zuheir Alshehabi

**Affiliations:** 1 Faculty of Medicine, Cancer Research Center, Tishreen University, Latakia, Syria; 2 Department of Thoracic Surgery, Tishreen University Hospital, Latakia, Syria; 3 Department of Pathology, Cancer Research Center, Tishreen University, Latakia, Syria

**Keywords:** Endobronchial metastasis, Clear renal cell carcinoma, Lobectomy, Case report

## Abstract

**Background:**

Endobronchial Metastasis from extrathoracic tumors is a rare neoplasm that accounts for approximately 1.1% of total endobronchial malignancies. The most common primary tumors associated with EBM are from the colorectal, breast, and kidney regions. Although it represents a late manifestation in the context of tumor progression, it can rarely antedate the diagnosis of the primary tumor.

**Case presentation:**

A 67-years-old male was referred from another city hospital to our thoracic surgery department due to a 4-months history of hemoptysis and productive cough. A chest X-ray and computed tomography scan showed a soft-tissue mass within the left main bronchus and atelectasis of the anterior segment of the left upper lobe. Furthermore, a flexible bronchoscopy revealed a hypervascular lesion occluding completely the left upper lobe bronchus. The patient underwent lobectomy and pathological examination suggested endobronchial metastasis from clear cell renal cell carcinoma. A second computed tomography scan of the abdomen and pelvis showed a well-defined mass arising from the lateral aspect of the right kidney; therefore, the patient underwent right radical nephrectomy three weeks later and pathology confirmed the diagnosis of clear renal cell carcinoma with endobronchial metastasis.

**Conclusion:**

Despite its rarity, physicians should consider the possibility of endobronchial metastases in the setting of endobronchial lesions. Proper diagnostic approaches should also be considered to rule out the potential of asymptomatic extrathoracic neoplasms. In this manuscript, we aimed to report a rare case -the first from Syria to our knowledge- of an endobronchial metastasis that preceded the diagnosis of renal cell carcinoma. Importantly, we reviewed the existing literature and discussed the diagnostic and treatment approaches.

## Background

Endobronchial metastasis (EBM) from extrathoracic tumors is a very rare neoplasm that accounts for approximately 1.1% of total endobronchial malignancies [1].

King and Castleman were the first to report the frequency of EBM from extrathoracic tumors. The most common primary tumors associated with EBM are from the colorectal, breast, and kidney regions [2].

Bronchoscopically, EBM is described as a visible lesion metastatic to the subsegmental or proximal central bronchus and histologically as a neoplasm that shares the same characteristics as the primary tumor [3].

It is essential to differentiate EBM from other malignancies because the treatment approaches differ. Histopathological examination is the mainstay for establishing the diagnosis [4].

Although EBM represents a late manifestation, it can be rarely detected before the diagnosis of the primary tumor [1].

Herein, we present a rare case of a Syrian male with an EBM that antedated the diagnosis of renal cell carcinoma.

## Case presentation

**Fig. 1:**
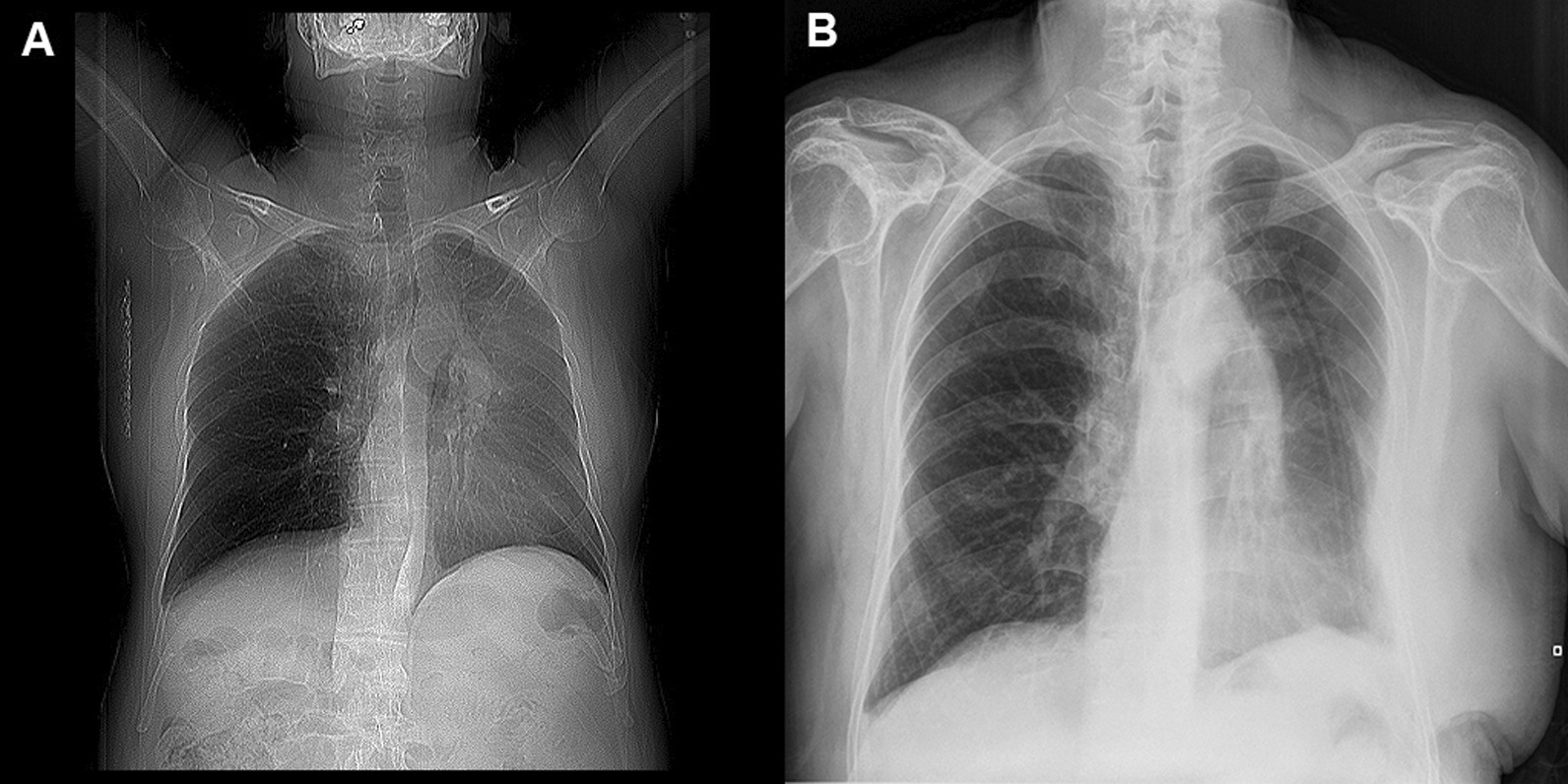
**A** Preoperative X-ray of the chest showing increased density of the left lung field. **B** Postoperative X-ray of the chest showing increased density in the lower zone of the left lung field resulting from left upper lobectomy

**Fig. 2:**
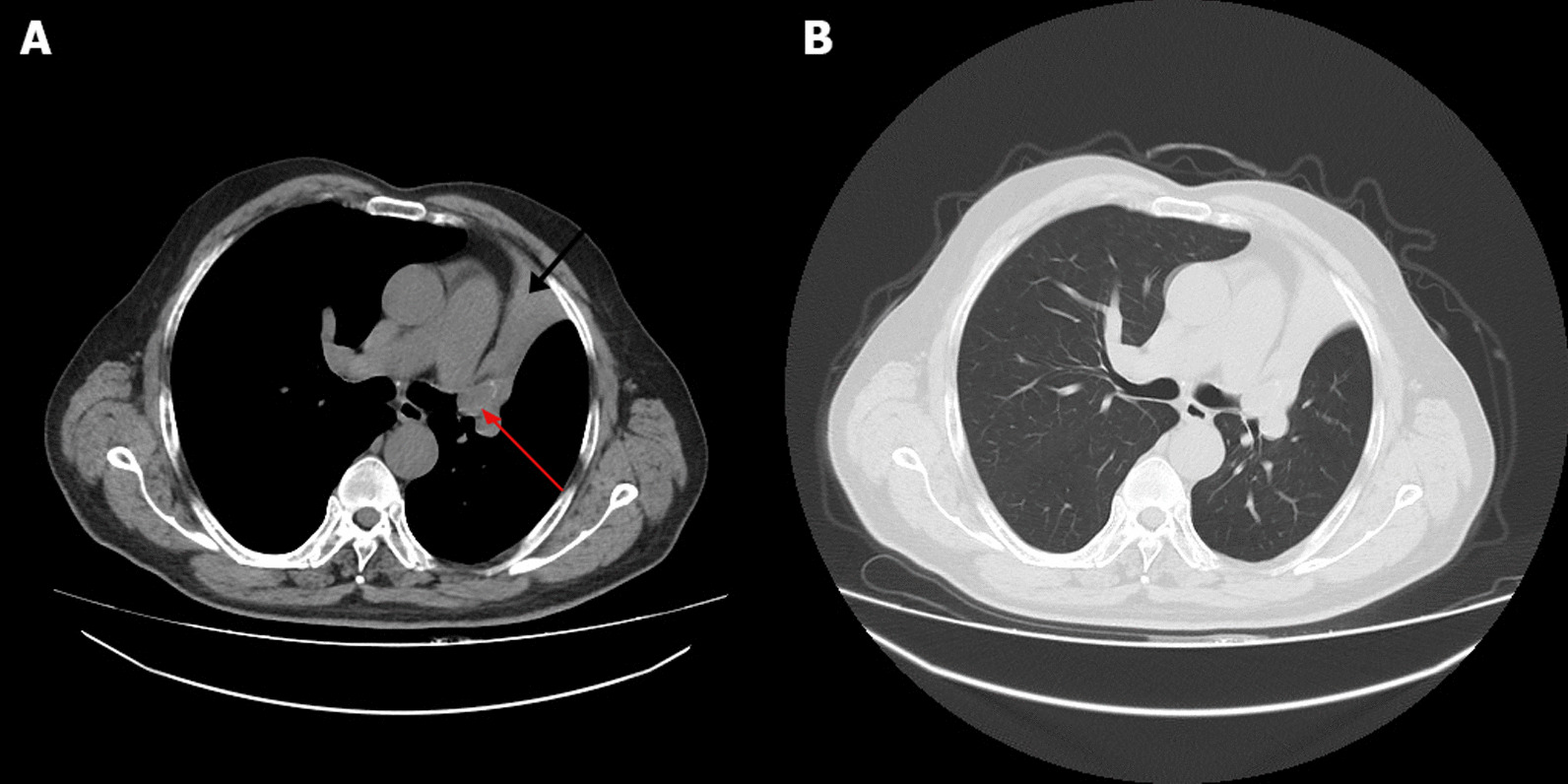
**A** Axial chest CT scan revealing soft-tissue mass measuring approximately (23 × 17) mm within the left main bronchus (red arrow) and atelectasis of the anterior segment of the left upper lobe (black arrow). **B** CT scan with chest window showing no lung parenchyma involvement or abnormalities

**Fig. 3:**
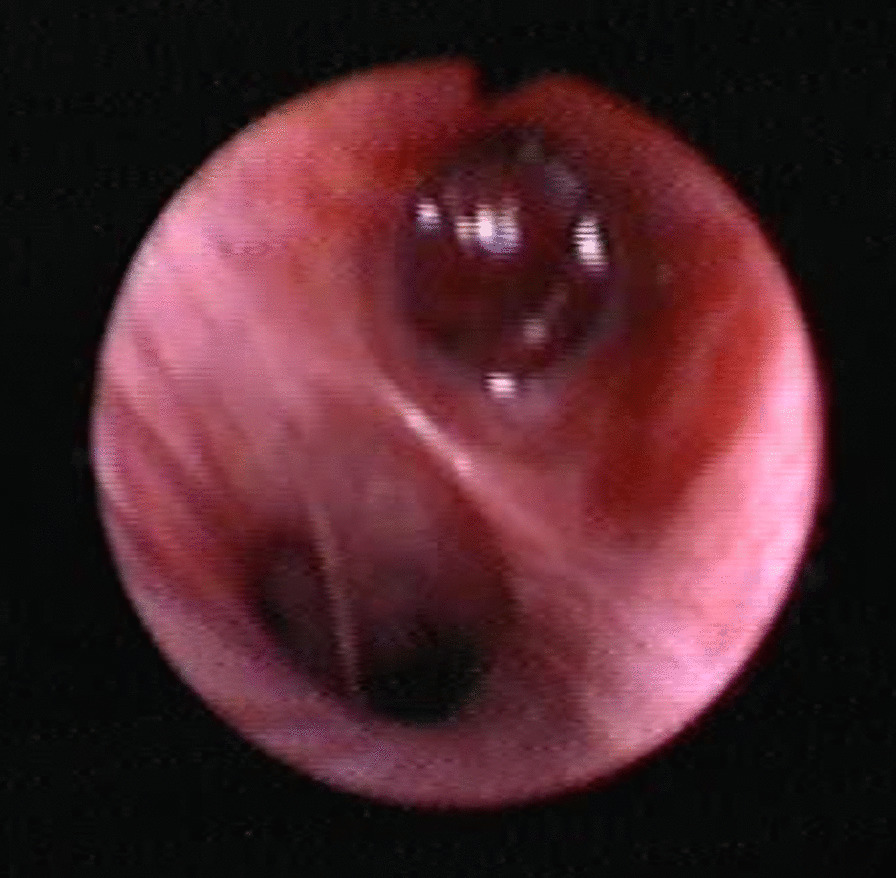
Bronchoscopy examination showing a hypervascular mass occluding the left main bronchus

**Fig. 4:**
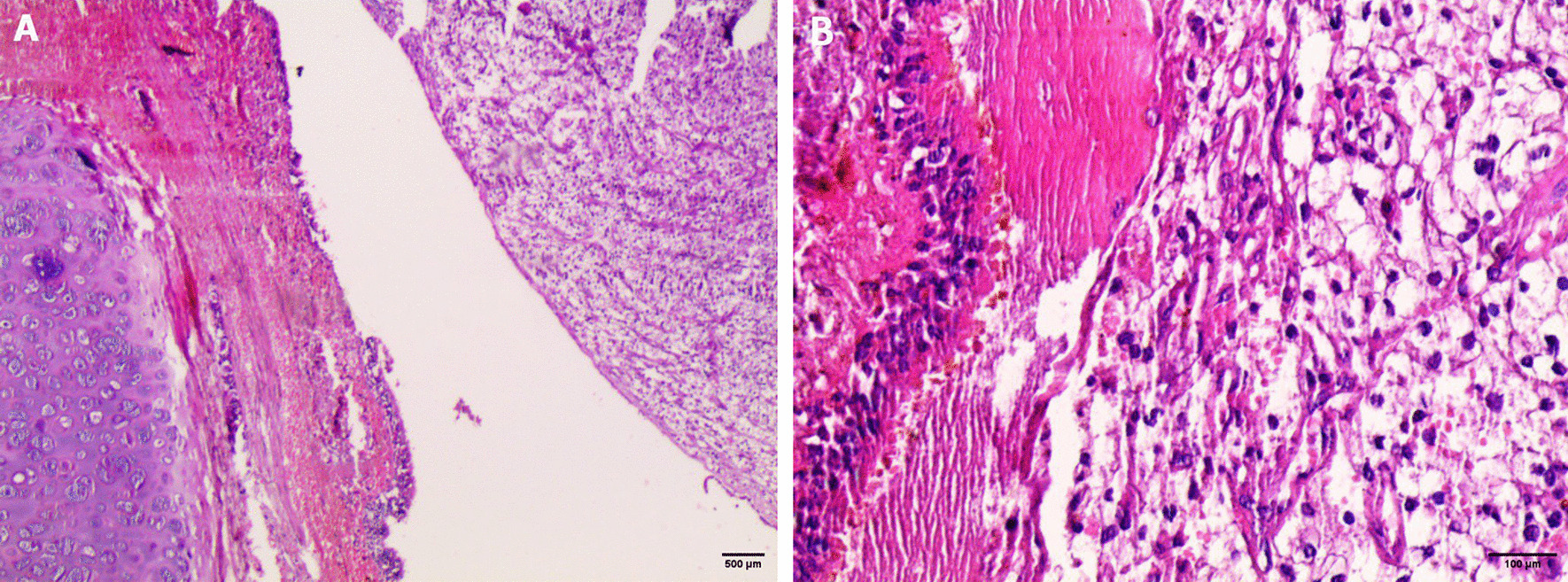
Pathological examination of the endobronchial lesion revealing abundant cells with clear cytoplasms and hyperchromatic nuclei **A** (hematoxylin and eosin [H&E] stain original magnification ×40, **B** hematoxylin and eosin [H&E] stain original magnification ×200). Pictures were taken by Nikon Eclipse Ni Upright microscope with CFI60 infinity optics, Digital Camera TP3100 CMOS 3.1 MPixels C-Mount at a resolution of 96 dpi and processed in Adobe Photoshop. No downstream processing was utilized

**Fig. 5:**
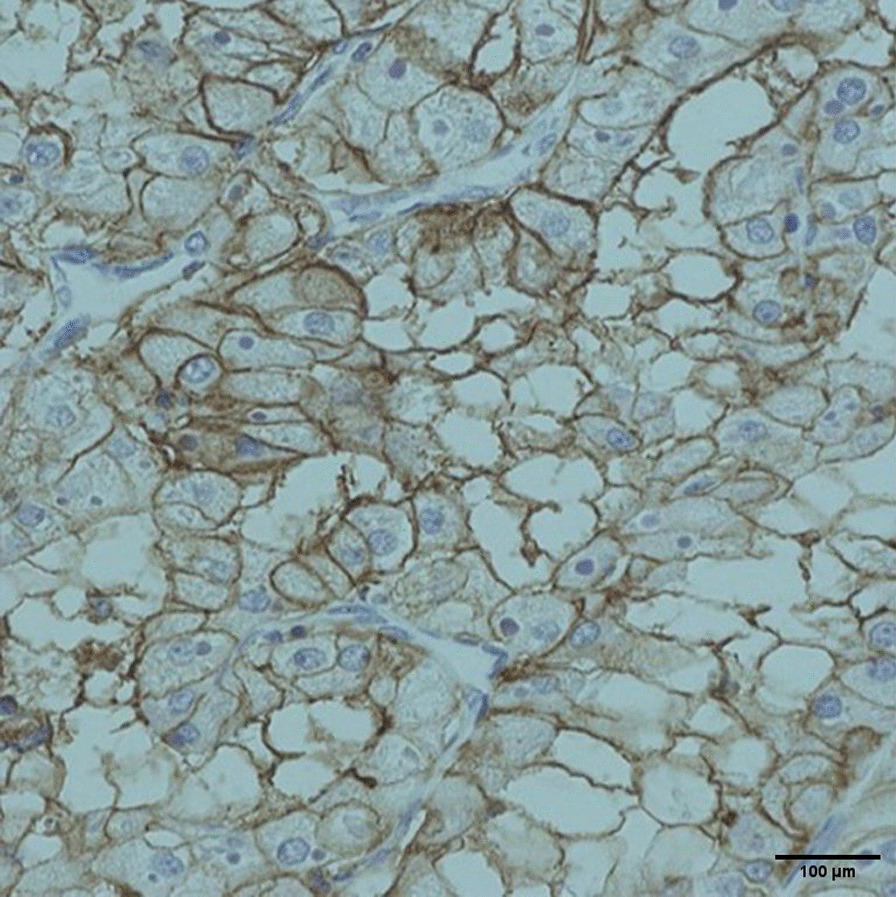
Immunohistochemistry of the endobronchial neoplasm showing positivity for CD10. (Original magnification × 200) Picture was taken by Nikon Eclipse Ni Upright microscope with CFI60 infinity optics, Digital Camera TP3100 CMOS 3.1 MPixels C-Mount at a resolution of 96 dpi and processed in Adobe Photoshop. No downstream processing was utilized

**Fig. 6:**
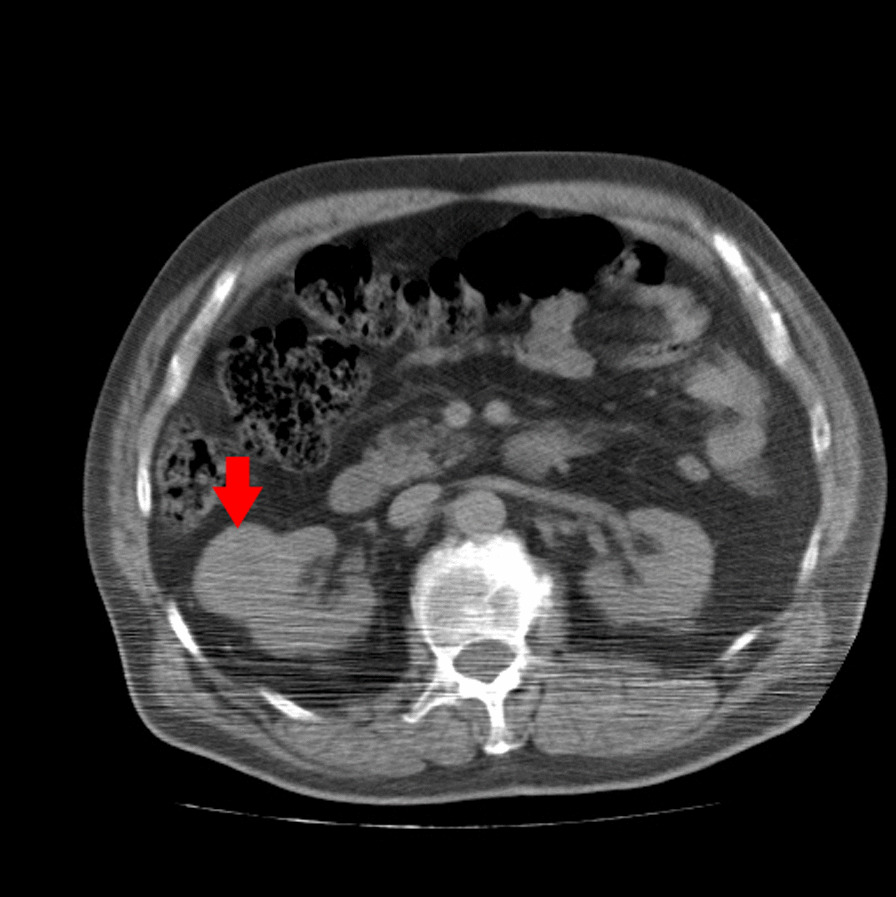
Axial computed tomography of the abdomen and pelvis revealing well-defined mass measuring approximately (45 × 40) mm arising from the lateral aspect of the right kidney

**Fig. 7:**
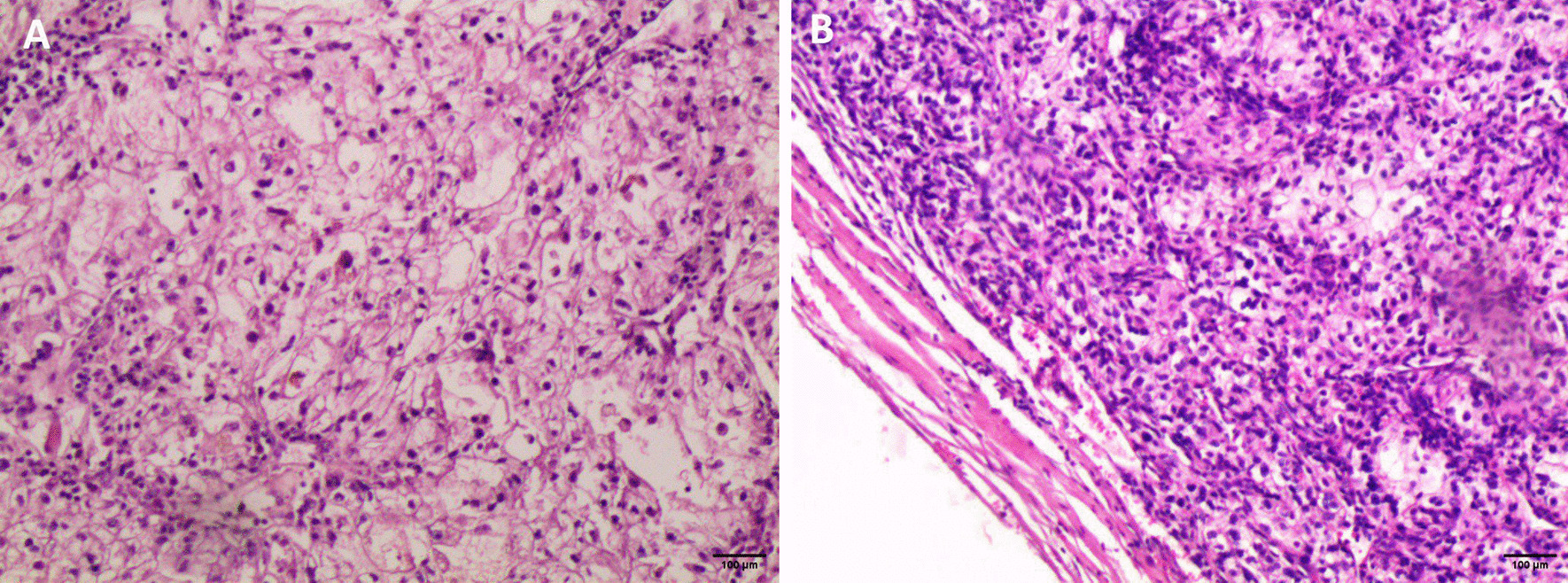
Pathological examinations of the renal mass **A** showing abundant cells with clear cytoplasms and hyperchromatic nuclei identical to the endobronchial lesion (hematoxylin and eosin [H&E] stain original magnification × 100). **B** No evidence of renal capsule invasion (hematoxylin and eosin [H&E] stain original magnification × 100). Pictures were taken by Nikon Eclipse Ni Upright microscope with CFI60 infinity optics, Digital Camera TP3100CMOS 3.1 MPixels C-Mount at a resolution of 96 dpi and processed in Adobe Photoshop. No downstream processing was utilized

A 67-years-old Syrian male was referred from another city hospital to our thoracic surgery department due to 4-month history of hemoptysis and productive cough. The patient was a non-smoker and non-alcoholic. There was no history of traveling. His medical and family history were unremarkable apart from HTN. The symptoms were not associated with fever, chest pain, dyspnea or weight loss. Physical examination revealed normal breath sounds in both lung fields. The abdomen was soft and non-tender with no palpable masses. Laboratory tests showed HGB 11.7 g/dL, RBC count 4.31 × 10^6^/μl and WBC count 12.7 × 10^3^/μl. A chest X-Ray and unenhanced computed tomography (CT) of the chest were ordered and demonstrated a soft-tissue mass within the left main bronchus and atelectasis of the anterior segment of the left upper lobe (Figs. 1,2). Subsequently, a flexible bronchoscopy was carried out for further assessment and revealed a hypervascular lesion occluding thoroughly the left upper lobe bronchus (Fig. 3). Biopsies of the lesion could not be obtained due to severe hemorrhage. The lesion was initially suspected as carcinoid. Therefore, the thoracic surgeon performed an urgent open left upper lobectomy to control the bleeding and establish the diagnosis. Pathological assessment of the mass tissue exhibited abundant cells with clear cytoplasms and hyperchromatic nuclei (Fig. 4). Subsequent immunohistochemistry showed positivity for CD10 (proximal tubular marker) (Fig. 5). Based on these findings, the diagnosis of endobronchial metastasis from clear renal cell carcinoma was suggested. Consequently, a CT scan of the abdomen and pelvis was ordered and revealed a well-defined mass measuring approximately (45 × 40) mm arising from the lateral aspect of the right kidney (Fig. 6). Therefore, the patient underwent a right radical nephrectomy three weeks later. Pathological examinations of the renal tumor showed abundant cells with clear cytoplasms and hyperchromatic nuclei without invasion of the renal capsule (Fig. 7). Based on the detailed microscopic findings along with radiological correlation, the patient was diagnosed with clear cell renal cell carcinoma with endobronchial metastasis (stage IV: T1N0M1) and was classified as having an intermediate prognosis according to the International Metastatic RCC Database Consortium (IMDC system).

The patient is currently well and is being monitored with CT scan every 6 months to detect potential metastases. No chemotherapy or radiotherapy was given to the patient.

## Discussion and conclusion

Although lungs are a frequent metastatic site for extra-thoracic tumors, it is rare to encounter metastases in the tracheobronchial tree [5]. EBM can occur at any airway level, however, its predilection is still uncertain.

A series of 174 cases by (Marchionni et al.) showed that EBM affects mostly the right side [6]. Similarly, Kiryu, et al. [3] reported in their series of 16 cases that EBM tends to involve the right lung in (80%) of cases. This is in contrast with Sørensen [7] who reported in his study that the prevalence of EBM was even in both right and left lungs (41%) . In our case, EBM occurred on the left side.

The clinical manifestations of EBM depend mainly on the size of the lesion and its location. The commonest symptoms are cough, hemoptysis, and dyspnea [2], which is consistent with our case. However, asymptomatic patients were previously reported by Kiryu et al. [3], Heitmiller et al. [8], Poe et al. [9] and Lee et al. [10].

Averagely, EBM is diagnosed (65.3) months after the diagnosis of the primary tumor, whereas our case was anachronous [3]. While searching the English medical literature, we found that only 8 cases, in which EBM antedated the diagnosis of renal tumor have been reported since 1972 (Table 1).

**Table 1:** Reported cases of EBM antedating the diagnosis of renal tumor since 1972

	Author name	Year	Respiratory Symptoms	Radiological Findings
1	Weintraub and Scully [12]	1972	Productive cough Hemoptysis	Left lower lobe posterior portion consolidation Right lung base medially infiltration
2	Braman and Whitcomb. (case 4) [13]	1975	Nonproductive cough Dyspnea	Right hilar mass
3	Bourke et al. [14]	1989	Cough	Right lower lobe infiltration
4	Kaneko et al. [15]	2003	Cough	Right upper lobe atelectasis Localized pleural effusion Right upper lobe bronchus polypoid mass
5	Marchioni et al*.* [6]	2014	N/A	
6			N/A	
7			N/A	
8			N/A	

EBM does not necessarily indicate a poor prognosis compared to the primary tumor without EBM. The prognosis is based upon the histological type of the primary tumor, the existence of metastases in other sites, the involvement of hilar or mediastinal lymph nodes, and complete surgical resection [4, 11].

Regarding the developmental modes of EBM, Kiryu et al. [3] proposed 4 types: type I is defined as direct metastasis to the bronchus whereas type II is bronchial invasion by a parenchymal lesion. Type III is bronchial invasion by mediastinal or hilar lymph node metastasis while type IV is the extension of peripheral lesions along the proximal bronchus [3].

Nevertheless, Akoglu [2] accepted in their retrospective study all EBM associated with parenchymal lesion as type II because it is difficult to distinguish type II and type IV, and considered endobronchial invasion with lymphangitis carcinomatosa as Type IV [2].

EBM can mimic other malignancies such as bronchogenic tumors and primary pulmonary tumors, so it is important to distinguish EBM from such malignancies [11].

The diagnostic procedures vary and include bronchoscopy with biopsy, surgical biopsy, bronchial brushing, CT, X-rays, FDG-PET, EBUS-TBNA, and bronchoalveolar lavage [4].

Bronchoscopy with biopsy is the most efficient method for evaluating intraluminal lesions. Yet its diagnostic yield in evaluating EBM is low due to the high false-negative rate.

In comparison, surgical biopsy has a higher yield but it is less preferable as it is more invasive and more expensive [4].

In our case, biopsies of the lesion could not be obtained due to severe hemorrhage.

Another diagnostic modality is bronchial brushing, which is an acceptable method with a reported sensitivity (94%) for non-hematologic metastases and an overall sensitivity (85%). However, this result was built on a single study conducted by (Kenji Ikemura) and further studies on its efficiency are required [16].

As for the remaining methods, they are considered secondary in evaluating EBM [4].

Atelectasis, hilar mass and multiple nodules are the most frequent findings on chest radiography. However, these manifestations are not specific to EBM since they can be seen in other diseases [7].

When bronchoscopy is not possible -due to cardiorespiratory failure or recent myocardial infarction-, CT can provide valuable information in evaluating the tracheobronchial tree and scan for other malignancies. EBM appears on CT as a polypoid, finger-glove shape or thickening lesion of the bronchus wall [17, 18]. Nevertheless, CT is not always able to demonstrate intraluminal lesions [2].

FDG-PET scan is a useful imaging modality for scanning and monitoring tumors and metastasis, yet it is not recommended in the diagnosis and staging of RCC as its role is still unclear. FDG PET/CT can be considered in RCC in postoperative surveillance when conventional imaging is not conclusive [19].

It is difficult to establish the diagnosis based only on the clinical and radiographical manifestations; thus, the definitive diagnosis requires microscopic examinations. Confirming the diagnosis is based on the comparison of both EBM and primary tumor specimens as they both share the same histopathological features.

Unfortunately, for the management of EBM, there is no well-established guideline. The management plan should be individualized based upon the site of the lesions, features of the primary tumors, finding of other metastatic sites, and the patient’s status [3, 4].

The main management choices are surgery, chemotherapy, radiotherapy, immunotherapy, and endobronchial therapies.

Radical surgery is recommended as the most efficient procedure for curing solely EBM, especially in the early stages. Other surgical approaches such as pneumonectomy and lobectomy can be useful in confined lesions [20]. When surgical resection of EBM is unattainable, lobectomy can be suitable in patients with a large localized lesion with an adequate pulmonary reserve (FEV1 > 1.5L). Moreover, severe hemorrhage can be encountered in hypervascular lesions like renal cell carcinoma and in such cases, bronchial artery embolization (BAE) or urgent lobectomy should be performed [4]. Unfortunately, BAE procedure is not available in our institution. Therefore, lobectomy was the best approach in our case to control the bleeding and remove the lesion as surgery was technically feasible.

When surgery is not possible, chemotherapy and external radiotherapy may be considered, especially in localized occluding lesions. However, they are limited of efficacy in RCC metastases, as they do not respond well to such modalities [21, 22].

On the other hand, immunotherapy played an important role in the management of advanced RCC in the last decade. Sunitinib used to be the standard first-line treatment; however, the combination of Axitinib (VEGFR inhibitor) with Pembrolizumab (immune checkpoint inhibitor) has become recently preferable as the first-line regimen in stage IV ccRCC patients according to the NCCN Clinical Practice Guideline v3.2022 [23].

A study by Rini et al. [24] showed longer overall survival, progression-free survival and a higher objective response rate of Axitinib with Pembrolizumab over Sunitinib.

Many palliative modalities can be also contemplated in the late stages of the tumor progression. These modalities include endobronchial therapies such as cryotherapy, laser therapy, brachytherapy, diathermic snares, intratumoral ethanol injections, argon plasma coagulation (APC), and stents [4, 22].

Cryotherapy is the application of near-freezing temperatures and the removal of abnormal lesions. (Eaton, Donna et al.) recommended cryotherapy as the first-line palliation modality in patients with symptomatic EBM. In their study on 35 patients, 30 patients (85%) showed major relief of their symptoms [25].

APC is another effective modality that uses argon gas to control hemorrhage from lesions. It can be also used in critical obstructions due to its immediate debulking results.

In their retrospective study, Rodolfo C. Morice and his colleagues applied the APC modality to 14 patients with metastatic endobronchial lesions and reported the resolution of bleeding in all patients with a significant reduction of the obstructive lesions sizes [26].

Although APC provides immediate relief, cryotherapy is considered safer as it does not affect the airway cartilages [27, 28].

Moreover, intratumoral ethanol injections can be helpful in debulking EBM and should be considered when ablation modalities such as (APC) fail [29].

Other less used endobronchial modalities are intraluminal brachytherapy, stents and laser therapy.

Intraluminal brachytherapy is reserved for diffused distal airway diseases in which other modalities are difficult to use [27]. Stranzl et al. [30] applied fractionated intraluminal high-dose-rate 192 Iridium to 11 patients with EBM and reported the alleviation of symptoms in the majority of patients (73%).

As for the use of laser therapy and stents, they are rarely reported in managing endobronchial metastasis.

Mention should be made that the aforementioned modalities are not considered a good treatment as they do not cure EBM, but aim to relieve the symptoms and improve the patients’ quality of life.

In conclusion, EBM from extrathoracic tumors remains a challenging disease due to its rarity; hence, physicians must be aware and consider the possibility of EBM in the setting of endobronchial lesions. Proper diagnostic approaches should be also considered to rule out the potential of asymptomatic extrathoracic neoplasms.

In this manuscript, we reported a rare case of EBM antedating the diagnosis of RCC and reviewed the existing literature while discussing the diagnostic and therapeutic methods hoping to draw more attention to this underdiagnosed entity.

## Data Availability

Data and material are available on reasonable request from the guarantor and mentor of this study Prof. Alshehabi.

## Abbreviations

EBM: Endobronchial metastasis
ccRCC: Clear cell renal cell carcinoma
CT: Computed tomography
BAE: Bronchial artery embolization
APC: Argon plasma coagulation
